# Outcome at Six Months After Primary Percutaneous Coronary Interventions Performed at a Rural Satellite Center of Sindh Province of Pakistan

**DOI:** 10.7759/cureus.8345

**Published:** 2020-05-28

**Authors:** Syed F Mujtaba, Muhammad N Khan, Hina Sohail, Jawaid A Sial, Musa Karim, Tahir Saghir, Kiran Abbas, Moiz Ahmed, Nadeem Qamar

**Affiliations:** 1 Adult Cardiology, National Institute of Cardiovascular Diseases, Karachi, PAK; 2 Interventional Cardiology, National Institute of Cardiovascular Diseases, Karachi, PAK; 3 Cardiology, National Institute of Cardiovascular Diseases, Karachi, PAK; 4 Statistics, National Institute of Cardiovascular Diseases, Karachi, PAK; 5 Medicine, Jinnah Postgraduate Medical Centre, Karachi, PAK; 6 Medicine and Surgery, Sindh Medical College, Karachi, PAK

**Keywords:** angiography, acute myocardial infarction, culprit vessel, des, percutaneous coronary intervention, pci

## Abstract

Introduction

Primary percutaneous coronary intervention (PPCI) is now a well-established treatment of acute ST-elevation myocardial infarction (STEMI). For the first time in Pakistan, various off-site satellite centers are established to perform PPCI 24-hours. Our population mainly resides in the rural area with low literacy rate and poor socioeconomic conditions. The majority of the patients who are presented in the satellite center had either never received any long-term treatment plan or were non-compliant to their medication. The objective of this study was to determine the outcome of patients at six months who underwent primary PCI at a rural satellite center of Sindh, Pakistan.

Methods

This study was conducted at Larkana satellite center of National Institute of Cardiovascular Diseases, Karachi. Patients who underwent PPCI for STEMI from October 2017 to March 2018 were enrolled in the study. In case of death of the patient, data were obtained from the attendant of the deceased. Patients, on follow-up visits, were interrogated for post-procedure symptoms.

Results

A total of 271 patients were enrolled in the study. The mean age ± standard deviation of patients was 54.84 ± 10.64 years. The most common culprit artery was left anterior descending (LAD) artery with 161 (59.4%) patients, followed by right coronary artery (RCA) with 98 (36.2%) patients. Only 41 (15%) patients had a three-vessel disease, while 141 (52%) patients had single-vessel disease. On follow-up, 70 (25.8%) patients complained of chest pain grade II, 20 (7.4%) complained of shortness of breath (SOB) grade II, 44 (16.2%) complained of vertigo, and 16 (5.9%) complained of nonspecific weakness. The mortality rate of 6.3% (17) was observed after six months of PPCI. The mortality rate was found to be lower for patients with LAD disease (p = 0.036) and higher among patients with RCA as the culprit artery (p = 0.045). The mortality rate was significantly associated with the number of diseased vessels and the type of stent deployed.

Conclusion

Primary PCI, at a rural satellite center, has an overall positive outcome. Steps should be taken to provide free medication along with encouragement towards compliance of dual antiplatelet medication. Furthermore, the facility for subsequent procedures should be provided at the same set-up.

## Introduction

The gold standard treatment for acute ST-elevation myocardial infarction (STEMI) is the primary percutaneous coronary intervention (PPCI) [1-3]. Therefore, various offsite satellite centers in rural areas in the Sindh province of Pakistan have been established. An offsite satellite center is defined as a center with no facility to perform an emergency coronary artery bypass graft (CABG). The location of these satellite centers is such that no area is outside of a two-hour travel distance away. Percutaneous coronary intervention (PCI) is performed by experienced operators as per European guidelines in these centers, round the clock [4-6]. These centers are equipped with enough procedural facilities and expertise to run as a separate entity.

According to the European Society of Cardiology (ESC) and the American Heart Association (AHA) guidelines, an annual volume of at least 75 procedures at an institute performing at least 400 PCIs per year is recommended to maintain competency of a facility [7,8]. Regulation and maintenance of these satellite centers are the responsibility of the National Institute of Cardiovascular Diseases (NICVD), Karachi, which is the world’s largest PPCI center. No such facility was previously present in the rural areas of Pakistan. Majority of these areas have a low literacy rate and poor socioeconomic conditions.

Our study is unique in two aspects. Firstly, the setting of the study was a twenty-four hour open facility for PPCI. Secondly, most of our population belonged to the rural area. The majority of the patients who presented to the satellite center had either never received any long-term treatment plan or were non-compliant to their medication [9]. Most of the patients belonged to a poor socioeconomic class and were not able to afford the treatment cost of subsequent procedures including CABG. Therefore, the outcome may vary as compared to the studies from the first-world countries.

## Materials and methods

This study was conducted at Larkana satellite center of National Institute of Cardiovascular Diseases. Patients who underwent PPCI for STEMI from October 2017 to March 2018 were enrolled in the study. Demographic information and medical history of the participants were retrieved from the hospital records. Angiographic profile and procedural characteristics were also recorded. The researchers obtained the post-procedure outcome after six months of the PPCI from the patients during the follow-up visit. The researchers retrieved the information over a phone-call for those patients who missed their follow-up visits. Before data collection, all patients gave informed verbal consent to participate in the study.

The primary outcome of interest was all-cause mortality after six months of the PPCI. The secondary outcomes were stent thrombosis, stenosis, re-infarction, stroke, or emergency CABG surgery. Patients also complained about their other symptoms like angina, dyspnea, weakness, and vertigo on the follow-up visit. A predefined Pro-forma was used to record the demographic and clinical outcomes for each patient. The data were analyzed using Statistical Package for the Social Sciences (SPSS), version 21.0 (IBM Corp., Armonk, NY). Mean ± standard deviation (SD) or percentage (frequency) was used to present continuous data while a chi-squared test was applied to assess the association between outcome, demographic, and clinical characteristics. A probability value (p-value) of less than 0.05 was considered to be statistically significant.

## Results

A total of 271 patients participated in the study. Out of these, 234 (86.3%) were male and 37 (13.7%) were female. Mean age of the patients was 54.84 ± 10.64 years. Forty (14.8%) of them were under the age of 40 years, while 90 (33.2%) respondents were above the age of 60 years. We reported a strong-risk profile with diabetic, hypertensive, and obese patients. Demographic profile and baseline risk factors have been presented in Table 1.

**Table 1:** Demographic profile, baseline risk factors, and disease burden

Characteristics	Total (n = 271)
Age (years)	53.94 ± 11.47
Up to 40 years	40 (14.8%)
41 to 60 years	141 (52.0%)
Older than 60 years	90 (33.2%)
Gender	
Male	234 (86.3%)
Female	37 (13.7%)
Risk factors	
Diabetes mellitus	105 (38.7%)
Hypertension	177 (65.3%)
Smoking	112 (41.3%)
Obesity	18 (6.6%)

The most commonly localized culprit lesion was the left anterior descending (LAD) artery with 161 (59.4%), followed by the right coronary artery (RCA) for 98 (36.2%) patients. Nearly 15% (41) patients had a three-vessel disease, whereas, 52% (141) patients had a single-vessel involved. Fifteen (5.2%) patients were treated with plain old balloon angioplasty (POBA), whereas 49.1% (133) and 45.8% (124) patients were treated with drug-eluting stent (DES) and bare-metal stent (BMS), respectively. Disease burden, localization of culprit lesion, and type of stent deployed during the procedure are presented in Table 2.

**Table 2:** Disease burden, localization of culprit lesion, and type of stent deployed during the procedure LAD: Left anterior descending; RCA: Right coronary artery; CX: Circumflex artery; OM branch: Obtuse marginal branch; SVD: Single-vessel disease; 2VD: Two-vessel disease; 3VD: Three-vessel disease; DES: Drug-eluting stent; BMS: Bare-metal stent; POBA: Plain old balloon angioplasty.

Culprit Artery	
LAD	161 (59.4%)
RCA	98 (36.2%)
CX	10 (3.7%)
OM branch	4 (1.5%)
Diagonal	1 (0.4%)
Number of Vessels Involved	
SVD	141 (52%)
2VD	89 (32.8%)
3VD	41 (15.1%)
Type of Stent Deployed	
DES	133 (49.1%)
BMS	124 (45.8%)
POBA	14 (5.2%)

On follow-up, 70 (25.8%) complained of chest pain grade II, 20 (7.4%) complained of shortness of breath (SOB) grade II, 44 (16.2%) complained of vertigo, and 16 (5.9%) complained of nonspecific weakness. Outcomes after six months of the primary percutaneous coronary interventions (PPCI) are presented in Table 3. On analysis of outcome, no patient had a stent-related complication (in-stent restenosis), five (1.8%) underwent subsequent stage percutaneous coronary intervention (PCI), and two (0.7%) underwent coronary artery bypass grafting (CABG). Among other outcomes, three (1.1%) patients had a cerebrovascular accident (CVA), two (0.7%) had major surgeries, and five (1.8%) had other major complications. The mortality rate of 6.3% was observed after six months of the primary PCI.

**Table 3:** Outcomes after six months of the primary percutaneous coronary interventions (PPCI) ACE: Angiotensin-converting enzyme

Drug Compliance	
Aspirin	231 (85.2%)
Clopidogrel	229 (84.5%)
Beta-Blocker	225 (83%)
ACE inhibitor	197 (72.7%)
Statin	185 (68.3%)
Symptoms	
Chest pain (grade II)	70 (25.8%)
Shortness of breath (grade II)	20 (7.4%)
Vertigo	44 (16.2%)
Nonspecific weakness	16 (5.9%)
None	121 (44.6%)

Mortality rate after six months of the PPCI was found to be lesser for the patients with culprit LAD artery, 3.7% vs. 10%, p = 0.036, and higher among patients with RCA as culprit artery, 10.2% vs. 4%, p = 0.045. Mortality rate after six months of PPCI was found to be positively associated with the number of diseased vessels and the type of stent deployed. Mortality after six months of the PPCI by baseline characteristics is presented in Table 4.

**Table 4:** Mortality after six months of the primary percutaneous coronary interventions (PPCI) by baseline characteristics LAD: Left anterior descending; RCA: Right coronary artery; SVD: Single-vessel disease; 2VD: Two-vessel disease; 3VD: Three-vessel disease; DES: Drug-eluting stent; BMS: Bare-metal stent; POBA: Plain old balloon angioplasty.

Characteristics	Base	Outcome		P-value
	Frequency	Expired	Alive	
Age				
Up to 40 years	40	1 (2.5%)	39 (97.5%)	0.350
41 to 60 years	141	8 (5.7%)	133 (94.3%)	
More than 60 years	90	8 (8.9%)	82 (91.1%)	
Gender				
Male	234	14 (6%)	220 (94%)	0.620
Female	37	3 (8.1%)	34 (91.9%)	
Involvement of LAD artery				
Yes	161	6 (3.7%)	155 (96.3%)	0.036*
No	110	11 (10%)	99 (90%)	
Involvement of RCA				
Yes	98	10 (10.2%)	88 (89.8%)	0.045*
No	173	7 (4%)	166 (96%)	
Number of Vessels Involved				
SVD	141	4 (2.8%)	137 (97.2%)	0.001*
2VD	89	5 (5.6%)	84 (94.4%)	
3VD	41	8 (19.5%)	33 (80.5%)	
Type of Stent Deployed				
DES	133	5 (3.8%)	128 (96.2%)	0.029*
BMS	124	9 (7.3%)	115 (92.7%)	
POBA	14	3 (21.4%)	11 (78.6%)	

## Discussion

Our study reported a six-month mortality rate of 6.3%. This is in accord with another report from an offsite PPCI center in the Netherlands [10]. Another study showed a higher 30-day mortality rate of 7% [11]. Another study, conducted in Cairo showed much higher mortality of 12% at six months [12]. In our population, the mortality rate was expected to be higher than the other studies because of many factors. Due to costly medication, patients who undergo percutaneous coronary intervention do not comply with dual antiplatelet therapy. Subsequent procedures of either stage PPCI or CABG were advised to patients but, only a few patients gave consent.

Our patients mainly belonged to low socioeconomic status and were not well-educated. This impeded their fast recovery as they were less compliant with medication and surgical protocol. Dual antiplatelet is very important in the initial days postoperatively [13,14]. Guidelines recommend at least one year of dual antiplatelet therapy in acute coronary syndrome (ACS) patients [15]. Continuation of dual antiplatelet for at least 12 months is essential with DES implantation, otherwise, the risk of stent thrombosis and in-stent restenosis (ISR) increases [7,8,16,17].

In the present study, the compliance rate for dual antiplatelet therapy was 85% at six months of PPCI. One study has reported a compliance rate of 95% after acute myocardial infarction (AMI), which is much higher than our population [18]. Lower compliance to dual antiplatelet therapy can be due to financial constraints or due to improper counselling regarding the continuation of these drugs. Several studies have shown that low socioeconomic status and lack of awareness are the two critical factors, contributing to drug non-compliance [19,20]. Majority of our population belonged to a low socioeconomic background and were uneducated.

The immediate relief from distressing symptoms following PPCI disinclines the patients to stick to their regular medications, which have no short-term effect on their symptoms. The side effects of these drugs can easily cause them to become non-compliant. Majority of these patients had been advised for the subsequent procedures either of stage PCI or CABG. However, very few of the patients underwent the recommended procedures. This could also be due to the immediate relief in symptoms following primary PCI, making them less worried about their condition. Secondly, at the time of the study, CABG was not done at this setup. The patient would have to travel about 500 kilometres away for the subsequent procedures. The low socioeconomic class usually cannot afford the transportation cost from one city to the other.

Our study showed that patients with three-vessel disease had higher mortality as compared to patients with one-vessel disease. This was in accord with other studies [21-24]. In spite of being advised, the majority of these patients did not undergo any subsequent surgery. Only seven patients underwent CABG and stage percutaneous coronary intervention (PCI). This finding was similar to other studies where the prognosis was better in total revascularization rather than culprit vessel PCI only [25-27]. Our study showed that patients with a culprit artery other than the LAD artery had higher mortality. Even without any subsequent procedures, the patients involving the LAD artery remained symptom-free. We reported an increased mortality rate in patients with the right coronary artery (RCA) disease.

This can be a reflection of the same trend that once RCA is stented, patients even with two- or three-vessel disease did not undergo a subsequent procedure. In contrast, one of the studies reported poorer outcomes with the involvement of the LAD artery [28]. We also reported higher mortality in patients in which isolated POBA was done. We conducted POBA mostly in the setting of a diffuse-disease, where the aim was to restore blood flow to the heart. POBA was only preferred in very critical situations. Therefore, the average mortality was high.

## Conclusions

An overall good outcome was observed at six months after primary percutaneous coronary interventions (PPCI) at a rural satellite center in Pakistan. The most important factor resulting in an unsatisfactory outcome was the non-compliance of patients with the dual antiplatelet therapy or refusal to undergo a subsequent surgical procedure as per advice. The low socioeconomic status with a poor educational background was the biggest hindrance faced during the study. Another critical factor influencing the outcome was the involvement of a three-vessel disease with a noticeably higher mortality rate in comparison to patients with a one-vessel disease. Free of charge medication and proper counselling sessions should be provided to patients to guide them about the consequences of not availing the dual antiplatelet therapy or other subsequent surgical procedures. Also, a facility for subsequent procedures of either stage PCI or CABG near the satellite center should be considered to facilitate the low socioeconomic class.
